# Psychometric properties of the survey work-home interaction nijmegen in Argentinian population

**DOI:** 10.3389/fpsyg.2022.876025

**Published:** 2022-07-18

**Authors:** Elena Lucía Colasanti, Estanislao Castellano, Lucas Lapuente, Luciana Sofía Moretti, Leonardo Adrián Medrano

**Affiliations:** ^1^Secretaría de Investigación y Transferencia Científica, Universidad Siglo 21, Córdoba, Argentina; ^2^Pontificia Universidad Católica Madre y Maestra, Santiago de los Caballeros, Dominican Republic

**Keywords:** reliability, validity, wellbeing, work–family relationship, organizational psychology

## Abstract

Frictions between work and family life have increased during the COVID-19 pandemic, causing negative consequences on the mental health and quality of life of workers. Without validated instruments, it is not possible to determine the impact of Work-Family and Family-Work conflict. To date, no studies have been conducted to provide evidence of the validity and reliability of The Survey Work-Home Interaction Nijmegen (SWING; 22 items) in the population of Argentine workers. The SWING was administered to 611 Argentine workers of both sexes (73.6% female) aged between 18 and 70 years (M = 35.33; SD = 9.16) selected from a non-probabilistic accidental sampling. The confirmatory factor analysis showed satisfactory fit indices of the original four-factor model (χ2 = 647.073, gl = 203, CFI = 0.93, GFI = 0.92, NFI = 0.90, TLI = 0.92, RMSEA = 0.05, SRMR = 0.05, AIC = 557.9, BIC = 821.5). The level of reliability was acceptable (α between 0.68 and 0.86, ω = 0.79–0.89). The relationships of the subscale scores with the engagement and burnout variables were as expected according to previous studies. Having an instrument adequately adapted to the population of Argentine workers facilitates the development of studies aimed at evaluating the role of W-F or F-W interactions and their implications for health and productivity.

## Introduction

The balance between family and work life is increasingly difficult to maintain in today’s world (Vesga-Rodríguez, 2019). Frictions between work and family life have increased during the COVID-19 pandemic that led to workers being forced to use a telecommuting mode. This unplanned transition, abruptly determined by circumstances, has generated multiple consequences, such as increased negative emotions and perceived lower productivity at work (Howe and Menges, 2020).

The imbalance or conflict Work-Family and Family-Work (W-F/F-W) has significant negative consequences on mental health and quality of life of individuals. Thus, numerous studies highlight that W-F/F-W conflict is associated with higher burnout (e.g., Dyrbye et al., 2011; Chernyak-Hai and Tziner, 2016; Rhnima et al., 2016; Robinson et al., 2016; Smith et al., 2019), worse job satisfaction, and low professional self-efficacy (Shimada et al., 2010; Serenko et al., 2017; Jacobsen and Fjeldbraaten, 2018). Conversely, the balance between W-F/F-W has been associated with other positive aspects, such as workers’ engagement, psychological sense, and availability at work (Rothmann and Baumann, 2014; Queirós et al., 2016; Łaba and Geldenhuys, 2018).

Given the relevance of these phenomena, several scales have been developed (Kopelman et al., 1983; Netemeyer et al., 1996; Kelloway et al., 1999; Frone, 2000; Dolcos and Daley, 2009; DiRenzo et al., 2011). The Survey Work-Home Interaction Nijmegen (SWING, Geurts et al., 2005) is one of the most widely used instruments worldwide. It is a brief self-report questionnaire (22 items) that measures the interaction of both areas, discriminating the direction of the influence, whether W-F or F-W, as well as the quality of the influence, both positive and negative. This instrument was developed based on scientific evidence, especially the Effort-Recovery Theory (Meijman and Mulder, 1998). In the original study, the scale demonstrated a robust four-factor structure, which was replicated in different samples.

SWING validations have been carried out in different cultures: France (Lourel et al., 2005), Romania (Ispas and Iliescu, 2019), Japan (Shimada et al., 2018), Portugal (Pereira et al., 2014) and South Africa (Marais et al., 2009), Spain (Moreno-Jiménez et al., 2009), and Latin American population (Romeo et al., 2014). Satisfactory results were obtained in all cases (the alpha ranged from 0.72 to 0.90), and the original four-factor model was replicated without the need to modify the structure of the instrument (Table 1).

**Table 1:** Psychometric data of the SWING scale and its validations to other populations.

Validation	Reliability		Structure: number of factors
Original Study Holand (Geurts et al., 2005)	α = 0.72 to 0.85		4
South Africa (Marais et al., 2009)	α = 0.82 to 0.90		4
Japan (Shimada et al., 2018)	α = 0.75 to 0.86		4
Portugal (Pereira et al., 2014)	α = 0.72 to 0.86		4
France (Lourel et al., 2005)	α = 0.73 to 0.84		4
Rumania(Ispas and Iliescu, 2019)			4
Spain (Moreno-Jiménez et al., 2009)	α = 0.77 to 0.89		4
Spanish talking countries (Romeo et al., 2014)	α = 0.85 to 0.90		4
Argentina (Gabini, 2017)	Coefficient of compound reliability = 0.81 and 0.82		2 Named: Work-Family Enrichment and Work–Family Conflict

Few psychometric studies were conducted in the Argentine population. The study by Gabini (2017) constitutes an approach of this valuable tool to the local population; however, the resulting scale and the process carried out may be questionable. In this study, the number of SWING items was reduced from 22 to 8, and the exploratory and confirmatory factor analyses showed only two dimensions. To date, no studies have been conducted to provide evidence of the validity and reliability of the SWING scale (22 items) in the population of Argentine workers. Having an instrument with adequate psychometric properties will allow the development of research on W-F/F-W conflict, as well as the evaluation of the impact of interventions aimed at improving workers’ wellbeing. For this reason, this study aims to evaluate the factor structure of SWING as well as the reliability of the scores and the construct of each dimension. In addition, we seek to gather valid evidence for its relationship with other variables by associating the SWING scores with burnout and engagement scores of Argentinian workers.

## Materials and methods

### Participants

The sample comprised 611 Argentinian workers of both sexes (73.6% women) aged between 18 and 70 years (M = 35.33; SD = 9.16) selected from a non-probabilistic accidental sampling. Of the participants, 36.1% reported being married, 38.2% single, 0.7% widowed, 0.8% divorced, and 24.3% cohabiting with their partner. 70.8% of the participants reported having 1–5 children (M = 1.56; SD = 1.35), with ages ranging from 0 to 40 years (M = 8.09; SD = 7.63). Of the workers, 71.8% worked full time and the remaining 28.2% worked part time (up to 6 h) at the time of data collection. Regarding the area of work: 37% worked in commerce, 9.1% in health, 2.4% in public administration, 6.1% in education, and the remaining 45.5% in other unspecified areas. The size of the sample was sought to exceed 300 cases to guarantee its adequacy to the expected statistical tests planned for this study, particularly in terms of factor analysis (Lloret-Segura et al., 2014; Ventura-León et al., 2020; Jordan-Muiños, 2021).

### Instruments

#### *Ad hoc* questionnaire of socio demographic data

Participants’ sex, age, marital status, existence of children, number of children, age of the youngest child, and work time (full-time/half-time) were inquired.

#### SWING scale

This scale is composed of 22 items that evaluate four types of interactions: positive work-family (for example: Having to organize your time at work has made you learn to better organize your time at home), negative work-family (for example: You have to work so much that you do not have time for your hobbies), positive family-work (for example: You have more self-confidence at work because your life at home is well organized), and negative family-work (for example: Problems with your partner/family/friends affect your work performance; Geurts et al., 2005). In other words, it differentiates between the quality and direction of interactions between the two spheres of life. The response options are Likert-type: Never (0), Sometimes (1), Often (2), and Always (3). The Spanish version adapted for the Spanish population by Moreno-Jiménez et al. (2009) was used for this study.

#### UWES engagement questionnaire

This scale has 17 items with three factors: vigor, dedication, and absorption (Utrecht Work Engagement Scale, adapted for workers in Córdoba, Argentina, Spontón et al., 2012). The responses are graduated and range from 0 = “never” to 6 = “always.” In the present study, the reliability level of this instrument was acceptable for all subscales (vigor α = 0.780, dedication α = 0.876, absorption α = 0.728, Oviedo and Campo-Arias, 2005).

#### Maslach burnout inventory-general survey

The validation made by Spontón et al. (2019) in the Argentine population was used (Schaufeli et al., 1996). The burnout and cynicism scales were used, each composed of four items with graduated response options (from 0 = “never,” to 6 = “always”). In this study, the level of reliability of these scales was adequate, with results of α = 0.756 for exhaustion, and α = 0.783 for cynicism.

### Procedure

The *ad hoc* Sociodemographic Data Questionnaire and the SWING Scale were administered to the entire sample (*N* = 611) in their respective workplaces, after agreement with the authorities of the organizations and the workers. The questionnaires were administered in small groups (approximately 10 people) in the workplace by the authors of the research, who provided the instructions and all the necessary information related to the research. In all cases, written consent was obtained by means of a note clarifying the purpose of the study and guaranteeing the voluntary and anonymous nature of participation. Finally, a written report with the main results and specific recommendations for optimizing recovery levels in workers was provided to the institutions and companies that agreed to participate in the research. The research was approved by the Ethics Committee of the university where the research project was submitted. A subsample (*n* = 165) was additionally administered the UWES Engagement Questionnaire and the Maslach Burnout Inventory-General Survey (MBI-GS) scales for the criterion validity study.

### Statistical analysis

Data handling, descriptive statistics, and reliability analysis were computed with the IBM SPSS software version 25 (IBM Corporation, 2017). A descriptive exploration of the data was carried out, in which atypical and missing cases were searched for, and the distribution of the responses was analyzed.

Subsequently, the internal structure of the instrument was analyzed by confirmatory factor analysis (CFA) with the M*plus* program version 8.3 (Muthén and Muthén, 2019). The χ2, χ2 goodness-of-fit value of p, degrees of freedom (gl), comparative fit index (CFI), goodness-of-fit index (GFI), normalized fit index (NFI), unnormalized fit index (TLI), standardized root mean squared residual (SRMR), root mean square error of approximation (RMSEA), Akaike information criterion (AIC), and Bayesian information criterion (BIC) were used. It was considered as critical values for CFI and TLI cutoff value close to 0.95 (Hu and Bentler, 1999); 0.93 for GFI (Cho et al., 2020); 0.90 and 0.95 acceptable and optimal fit for NFI, respectively (Sideridis and Jaffari, 2021); 0.06 for RMSEA (Hu and Bentler, 1999); and 0.08 for SRMR (Cho et al., 2020) smaller value for BIC (Profillidis and Botzoris, 2018) and AIC (Lord et al., 2021).

The reliability of the instrument was then analyzed using Cronbach’s Alpha and the Omega coefficient (Viladrich et al., 2017; Hayes and Coutts, 2020; Kalkbrenner, 2021). Omega coefficient takes into account the ordinal nature of the data and, as such, it is recommended for Likert-type item scores (Viladrich et al., 2017). In order to provide common reference points with the previous literature, Cronbach’s alpha with the items treated as continuous was also computed and reported. According to George and Mallery (2003) reliability coefficients can be interpreted using the following guide: ≥0.90 excellent, ≥0.80 and <0.90 good, ≥0.70 and <0.80 acceptable, ≥0.60 and <0.70 questionable, ≥0.50 and <0.60 poor, and <0.50 unacceptable. We expect to obtain values between 0.90 and 0.80.

Finally, the relationships between the results obtained from the SWING Scale and the results obtained from the UWES Engagement Questionnaire and the MBI-GS scales were explored to verify concurrent validity.

### Ethical considerations

The present study was conducted following the ethical standards of Argentina, as well as the Universal Declaration of Ethical Principles for Psychologists [International Association of Applied Psychology and International Union of Psychological Science (IAAP and IUPsyS), 2008], the International Ethical Guidelines for Biomedical Research Involving Human Subjects [Organización Panamericana de la Salud and Consejo de Organizaciones Internacionales de las Ciencias Médicas (OPS and CIOMS), 2017], and the Declarations of the Interamerican Society of Psychology.

## Results

### Exploratory and descriptive analysis

Firstly, the initial exploration of the data was carried out attending to outlier cases, missing cases, mean, standard deviation, skewness and kurtosis. Sixty-three outliers (Z > 3 and <3.5) were detected and retained in the database because their presence did not markedly alter the distribution of the data. An outlier case of Z = 20 was also detected; this case was eliminated from the base because of its large deviation in relation to the rest of the data. No missing data above 5% were found; however, these were imputed by linear regression for the subsequent CFA tests. The data presented a normal distribution in all items with the exception of one item with skewness and kurtosis above the ±1.5 criterion (George and Mallery, 2003), namely, item 20 skewness = 1.72 and kurtosis = 2.17.

### Confirmatory factor analysis

**Figure 1:**
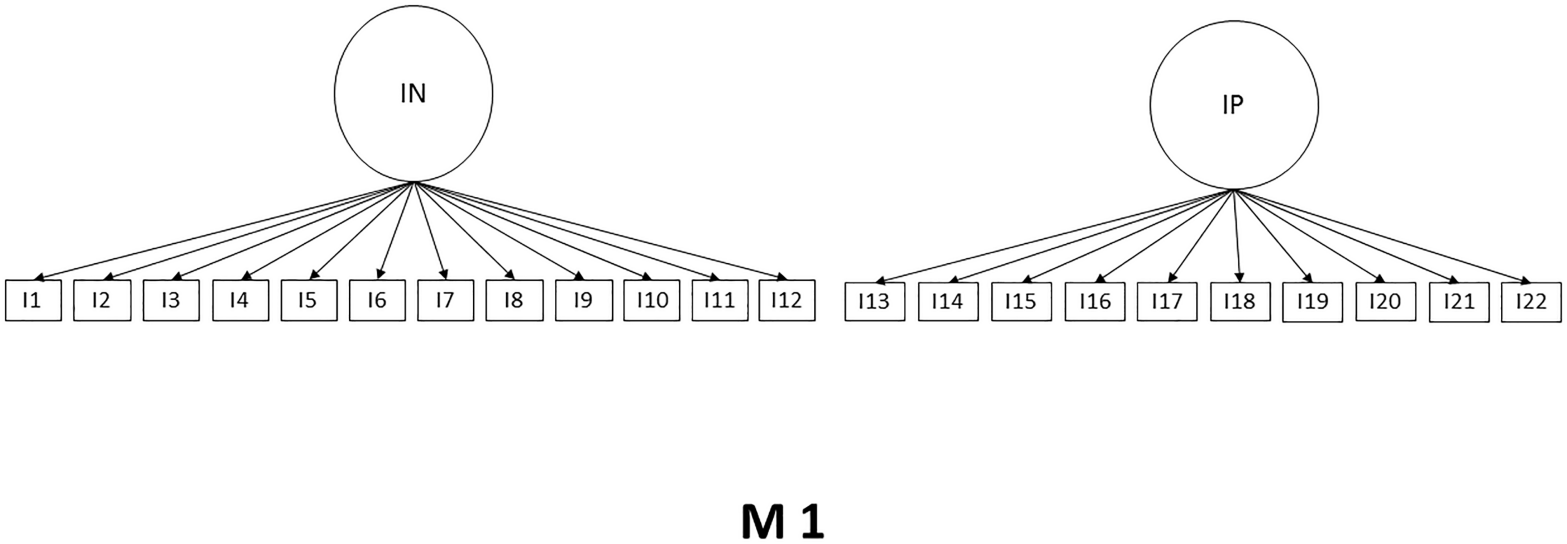
Model 1.

**Figure 2:**
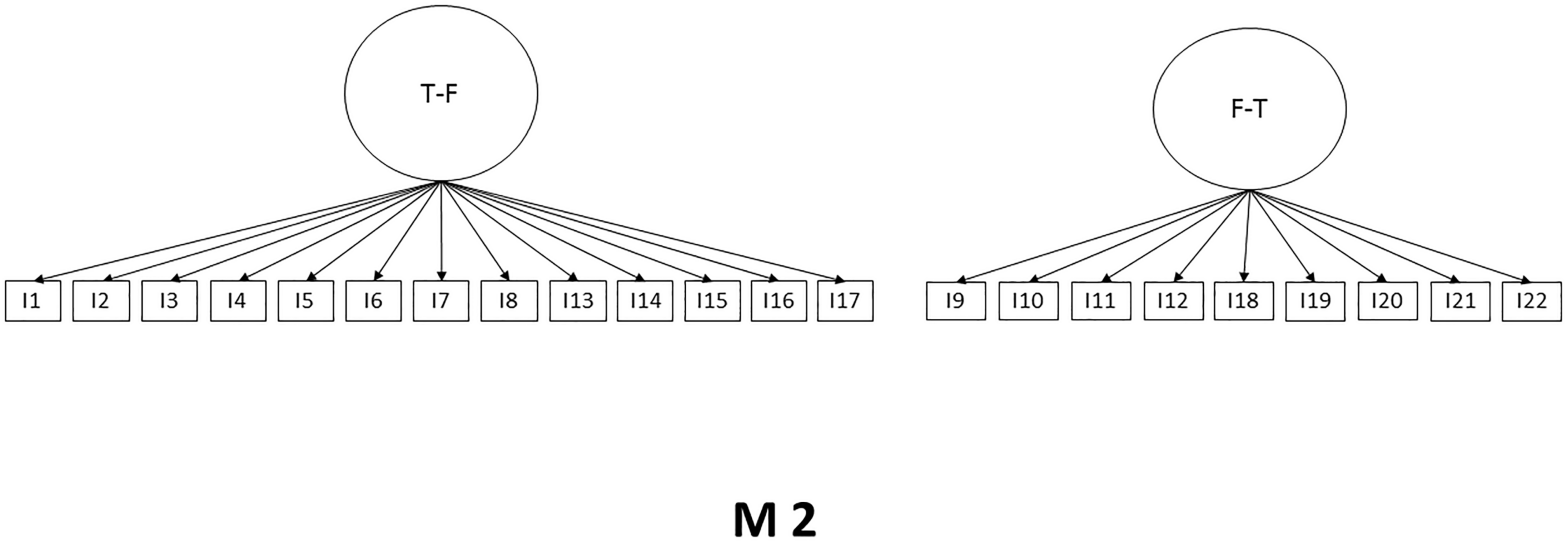
Model 2.

**Figure 3:**
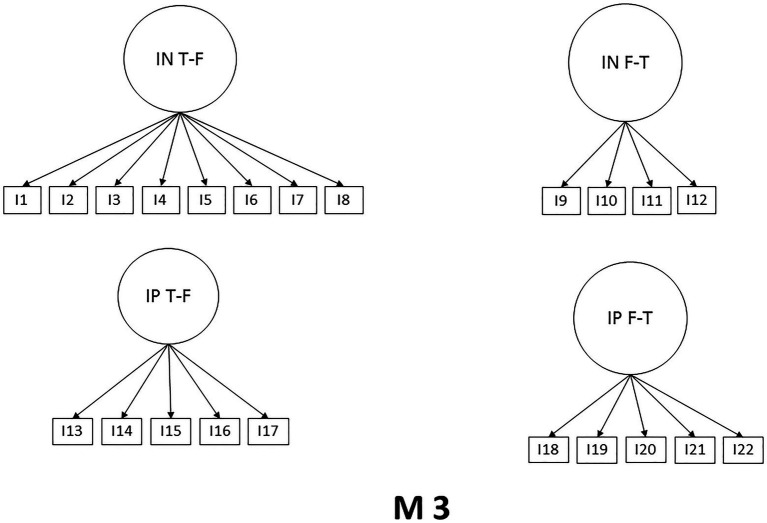
Model 3.

Considering the theoretical and psychometric background of the SWING scale, the CFA was performed. Three models with different numbers of factors were subjected to analysis. Model one (M1) shows a structure of two factors considering positive and negative interactions, model two (M2) also presents a two-factor structure of interactions family to work and work to family, while model three (M3) maintains the original structure of four-factor, the models are presented in Figures 1–3. The Maximum Likelihood Method was used, and multiple indicators were considered. It is observed that the original four-factor model (M3) is the one that presents a superior fit to the rest of the models tested. It should be clarified that the model still improves through three error correlations, as shown in M3r. The results are shown in Table 2.

**Table 2:** Fit indices for each specified model of the SWING scale in Argentine workers.

	Fit indices									
	χ^2^ (*p*)	*Df*	CFI	GFI	TLI	NFI	RMSEA	SRMR	AIC	BIC
M1	1332.273 (0.000)	208	0.76	0.79	0.73	0.72	0.09	0.83	1420.27	1613.59
M2	2412.127 (0.000)	208	0.52	0.66	0.47	0.50	0.13	0.17	2500.13	2503.65
M3	487.147 (0.000)	196	0.93	0.92	0.92	0.90	0.05	0.05	601.147	851.581
M3r	437.87 (0.000)	193	0.95	0.95	0.94	0.91	0.04	0.05	557.9	821.5

### Reliability analysis

The internal consistency of each subscale of the SWING was examined by means of Cronbach’s Alpha and the Omega coefficient. The results, presented in Table 3, are considered optimal and acceptable except for the α obtained in the *Positive Work-Family Interaction subscale*; however, the same yields satisfactory levels by using the ω coefficient.

**Table 3:** Internal consistency of the SWING subscales.

Subscale	α	ω
Work–Family negative interaction	0.864	0.894
Family–Work negative interaction	0.836	0.890
Work–Family positive interaction	0.686	0.794
Family–Work positive interaction	0.786	0.855

### Concurrent validity

Concurrent validity was then assessed by exploring the correlations between the results of the different SWING subscales, the UWES Engagement Questionnaire, and the MBI-GS scales. The results are in line with what is expected from the literature, as the negative interaction subscales correlated negatively with the engagement dimensions and positively with the burnout dimensions; on the contrary, the positive interaction subscales presented positive associations with the engagement variables and negative associations with the burnout variables. The results were mostly statistically significant and are shown in Table 4.

**Table 4:** Correlations between SWING subscales and engagement and burnout variables.

	Vigor	Dedication	Absorption	Exhaustion	Cynicism
Negative interaction W-F	−0.023	−0.073	0.168^*^	0.687^*^^*^	0.222^**^
Negative interaction F-W	−0.415^**^	−0.347^**^	−0.266^**^	0.260^**^	0.320^**^
Positive interaction W-F	0.209^**^	0.316^**^	0.175^*^	−0.217^**^	−0.332^**^
Positive interaction F-W	0.390^**^	0.378^**^	0.189^*^	−0.225^**^	−0.387^**^

* *p <* 0.05.

** *p <* 0.01.

In addition, SEM analyses were performed to verify the regression weights between the variables. The results show significant relationships of all subscales with some engagement and burnout variables (see Table 5).

**Table 5:** Regression weights.

			Estimate	S.E.	C.R.	*p*
Vigor	←	WFNEG	0.151	0.085	1.775	0.076
Dedication	←	WFNEG	0.077	0.11	0.699	0.484
Absorption	←	WFNEG	0.266	0.085	3.122	0.002
Exhaustion	←	WFNEG	0.708	0.065	10.902	***
Cynicism	←	WFNEG	0.093	0.078	1.189	0.235
Vigor	←	FWNEG	−1.37	0.225	−6.101	***
Dedication	←	FWNEG	−1.305	0.291	−4.485	***
Absorption	←	FWNEG	−0.953	0.226	−4.225	***
Exhaustion	←	FWNEG	0.349	0.172	2.029	0.042
Cynicism	←	FWNEG	0.772	0.207	3.736	***
Vigor	←	WFPOS	0.091	0.109	0.834	0.404
Dedication	←	WFPOS	0.357	0.141	2.531	0.011
Absorption	←	WFPOS	0.163	0.109	1.489	0.137
Exhaustion	←	WFPOS	−0.153	0.083	−1.832	0.067
Cynicism	←	WFPOS	−0.29	0.1	−2.897	0.004
Vigor	←	FWPOS	0.483	0.106	4.54	***
Dedication	←	FWPOS	0.486	0.138	3.526	***
Absorption	←	FWPOS	0.169	0.107	1.58	0.114
Exhaustion	←	FWPOS	−0.024	0.081	−0.298	0.766
Cynicism	←	FWPOS	−0.299	0.098	−3.062	0.002

*** <0.001.

## Discussion

Given the important consequences of positive and negative interactions between work and family life, valid and reliable assessment instruments are required for their evaluation. This is especially important given the context of the COVID-19 pandemic, which highlights the impact of these interactions on mental health. The aim of the present study was to evaluate the psychometric properties of the SWING scale for a population of Argentine workers. The results provide evidence of a solid internal structure, acceptable levels of reliability, and concurrent validity in accordance with the scientific literature.

It should be noted that the instrument allows differentiating not only the value of the interactions between positive and negative, but also the direction of these interactions, either W-F or F-W. The internal structure was corroborated by CFA, and indicators of a good fit to the replication of the original four-factor model (Geurts et al., 2005) were obtained without eliminating any item. These results are consistent with previous studies of adaptation to other populations (Lourel et al., 2005; Marais et al., 2009; Moreno-Jiménez et al., 2009; Pereira et al., 2014; Romeo et al., 2014; Shimada et al., 2018; Ispas and Iliescu, 2019) although they differ from the results reported by Gabini (2017) in Argentina, obtained through methodological processes different from those of the present study.

One aspect of interest is the use of residual error correlation in the CFA process to improve the fit indicators. This procedure improved the results of the present study and was also reported in the studies of Pereira et al. (2014) and Lourel et al. (2005). Relying on this background, it is possible to interpret that the lack of independence of the errors of these items could indicate the existence of common factors not specified by the model.

The reliability levels of the instrument were acceptable and optimal in this study. This is consistent with previous validations in other populations (Lourel et al., 2005; Marais et al., 2009; Moreno-Jiménez et al., 2009; Pereira et al., 2014; Romeo et al., 2014; Shimada et al., 2018; Ispas and Iliescu, 2019). The results improve with estimation using the omega coefficient, which is to be expected considering that Cronbach’s alpha is affected by the presence of uncorrected correlated errors (Viladrich et al., 2017).

The relationships between the results of the SWING scale and the burnout and engagement scales were mostly significant and consistent with other studies. Thus, burnout variables were positively associated with negative interactions either W-F or F-W, and negatively associated with positive interactions, coinciding with results from, among others, Chernyak-Hai and Tziner (2016), Dyrbye et al. (2011), Rhnima et al. (2016), Robinson et al. (2016), and Smith et al. (2019). For their part, engagement dimensions correlated positively with positive W-F and F-W interactions, and negatively with negative interactions; similar results were reported by Łaba and Geldenhuys (2018), Queirós et al. (2016), and Rothmann and Baumann (2014). However, the positive relationship between the results of the Negative W-F Interaction subscale and the scores obtained in absorption is striking since negative W-F interaction would be expected to hinder absorption at work, the role of absorption in this context should be further investigated.

Nevertheless, this study has some limitations that should be considered in future studies. On the one hand, the sample was non-probabilistic and it consisted mainly of women, which could have affected the measurement of some variables. Considering that there are contradictory antecedents in the Argentine population, it would be very important that further studies could analyze the stability of the psychometric properties of the SWING.

Since work-family relationships are crucial variables for individual, family, and organizational wellbeing, especially considering the changes generated by the COVID-19 pandemic, it is hoped that the validation of this instrument will contribute to and encourage the study of these and other related phenomena. Providing a reliable tool for professional practice in their diagnostic and health promotion processes. Having an instrument adequately adapted to the population of Argentine workers facilitates the development of studies aimed at evaluating the role of W-F or F-W interactions and their implications for health and productivity. On the other hand, the present work has practical implications since it provides a useful input for the identification of workers with W-F /F-W conflict and for the evaluation of interventions aimed at promoting a healthier work-life balance.

## Data availability statement

The raw data supporting the conclusions of this article will be made available by the authors, without undue reservation.

## Ethics statement

Ethical review and approval was not required for the study on human participants in accordance with the local legislation and institutional requirements. The patients/participants provided their written informed consent to participate in this study.

## Author contributions

ELC, EC, and LAM contributed to conception and design of the study. ELC and LL organized the database and performed the statistical analysis. ELC wrote the first draft of the manuscript. EC, LL, LSM, and LAM wrote sections of the manuscript. All authors contributed to the article and approved the submitted version.

## Funding

The authors declare that this study received funding from Santander Bank. The funder was not involved in the study design, collection, analysis, interpretation of data, the writing of this article or the decision to submit it for publication.

## Conflict of interest

The authors declare that the research was conducted in the absence of any commercial or financial relationships that could be construed as a potential conflict of interest.

## Publisher’s note

All claims expressed in this article are solely those of the authors and do not necessarily represent those of their affiliated organizations, or those of the publisher, the editors and the reviewers. Any product that may be evaluated in this article, or claim that may be made by its manufacturer, is not guaranteed or endorsed by the publisher.
